# Therapeutic Approaches to Tackle the Challenge of Depression That Is Resistant to Treatment–A Narrative Review

**DOI:** 10.1002/hsr2.70370

**Published:** 2025-01-22

**Authors:** Md. Rajdoula Rafe, Abdul Waris, Pranoy Saha

**Affiliations:** ^1^ Department of Neuroscience City University of Hong Kong Kowloon Hong Kong SAR China; ^2^ Department of Pharmacy Jagannath University Dhaka Bangladesh; ^3^ Department of Biomedical Sciences City University of Hong Kong Kowloon Hong Kong SAR China

**Keywords:** anti‐depressant, pharmacological, stimulation, therapeutics, treatment‐resistant depression (TRD)

## Abstract

**Background and Aims:**

The lack of therapeutic response characterizes treatment‐resistant depression despite undergoing at least two adequate monotherapy trials with medications from distinct pharmacologic classes. The inability to attain remission in patients diagnosed with major depressive disorder (MDD) is a significant issue of concern within public health. Therefore, the management of treatment‐resistant depression (TRD) poses significant obstacles for both patients and healthcare professionals. Our goal was to investigate the published literature concerning different options for treatment for TRD, including those that do not involve the use of medications.

**Methods:**

We thoroughly searched the literature in the Google Scholar, PubMed, and ScienceDirect databases to find publications relevant to our narrative review and extracted data from appropriate data. For this review, literary works written solely in English were chosen.

**Results:**

Ongoing research is being conducted to explore the treatment options for TRD, including pharmacological and nonpharmacological interventions. Pharmacological interventions include a wide range of therapeutic approaches, including but not limited to investigating innovative medications and strategies such as augmentation, switching, and combination therapies involving established and emerging drugs. Nonpharmacological interventions, including brain stimulation such as theta burst stimulation, deep brain stimulation, electroconvulsive therapy, repetitive transcranial magnetic stimulation, intermittent transcranial magnetic stimulation, and magnetic seizure therapy, as well as psychotherapeutic approaches, are being explored for the management of TRD in both present and future contexts.

**Conclusion:**

Researchers are dedicating significant resources to the aforementioned therapeutic interventions to advance the development of novel and efficacious treatment options for TRD and enhance our comprehension of the underlying disease. This review focused on looking at recent research concerning therapeutic interventions for TRD.

## 1 Introduction

Individuals, communities, and economies all bear the costs of depression, making it one of the top causes of disability worldwide [1]. Sadness, emptiness, or irritation, lasting at least 2 weeks, with accompanying physical and cognitive changes that impair functioning constitute depression [2]. Adolescence is a common starting point for depressive symptoms. However, clinical antecedents such as prolonged subthreshold depression symptoms or other mental or neurodevelopmental abnormalities are present in many patients before the first episode. Anxiety in early adolescence is the most common trigger for adult depression. An additional noteworthy observation is that the presence of chronic irritability during childhood serves as a prognostic indicator for the development of depression during adolescence [3, 4, 5].

Individuals diagnosed with major depressive disorder (MDD) or bipolar disorder commonly exhibit episodes characterized by a depressive state. Treatment‐resistant depression (TRD) is a distinct category within the diagnostic framework of MDD [6]. Individuals presenting with a depressive disorder that does not respond satisfactorily to appropriate treatment are commonly diagnosed with TRD. This particular form of depression is characterized by its resistance to conventional therapeutic interventions [7].

The definition of treatment‐resistant depression lacks consensus among scholars. However, a suboptimal response to a single sufficient trial of an antidepressant is indicative of a negative prognosis. Furthermore, the operationalization of TRD in clinical trials often involves an insufficient response to two or more treatments of appropriate dosage and duration during the current major depressive episode [8]. A notable proportion of individuals diagnosed with major depressive disorder exhibit an insufficient response to initial antidepressant interventions, resulting in what is referred to as treatment‐resistant depression. The presence of TRD has been observed to have connections with comorbid anxiety disorder, an increased risk of suicide, the presence of melancholic features, and a lack of response to an initial antidepressant. Additionally, individuals with TRD may have a body mass index (BMI) equal to or greater than 30 kg/m^2^, experience a depressive episode lasting longer than 2 months, be engaged in psychotherapy, exhibit sexual dysfunction, and have higher levels of depression severity [9, 10].

**Figure 1:**
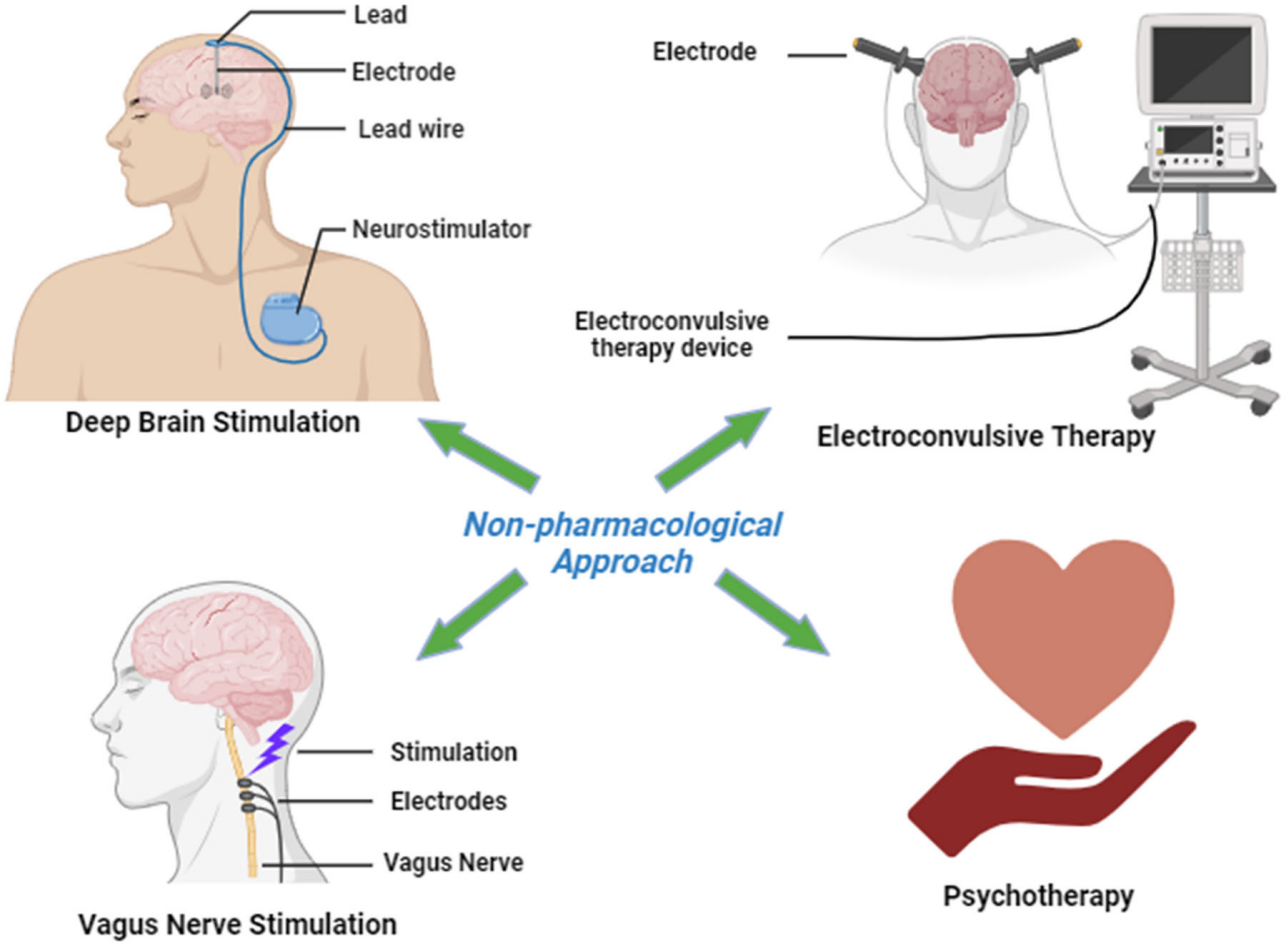
Some nonpharmacological interventions to deal with treatment‐resistant depression.

The subsequent therapeutic interventions for individuals experiencing TRD encompass various approaches (Figure 1). These include transitioning to an alternative antidepressant, employing a combination of multiple antidepressants, or supplementing an antidepressant with a nonantidepressant medication. Additionally, neuromodulation, such as diverse forms of brain stimulation (including electroconvulsive therapy, repetitive transcranial magnetic stimulation, magnetic seizure therapy, and deep brain stimulation [DBS], etc.) and psychotherapeutic strategies, are outlined (Table 1) [11, 12].

**Table 1:** Defining treatment approaches for the TRD.

Name of the strategies	Brief definitions	References
Augmentation	To boost the efficacy of a traditional antidepressant, “augmentation” involves the addition of medications that are not considered “antidepressants.”	[13]
Switching	When an initial antidepressant fails to work, switching to another medication can be helpful.	[14]
Combination	When two antidepressants are used together, or when one drug is used in conjunction with psychotherapy, the term “combination” is employed.	[15]
Complementary and alternative medicine	The use of unorthodox methods in conjunction with conventional treatment is known as “complementary” therapy, whereas the use of unorthodox methods in place of conventional treatment is known as “alternative” therapy.	[16]
Deep brain stimulation (DBS)	DBS entails the placement of a permanent neurosurgical implant in the brain to stimulate or inhibit a particular target. When activated from the outside, the implant sends pulses to a pulse generator in the chest wall, stimulating the area repeatedly.	[17]
Vagus nerve stimulation (VNS)	VNS uses a programmed neurostimulator to stimulate the left cervical vagus nerve.	[18]
Repetitive transcranial magnetic stimulation (rTMS)	rTMS is the name for delivering TMS in which many pulses are delivered at short intervals over the same location.	[19]
Electroconvulsive Therapy (ECT)	ECT is administered as a series of high‐frequency electrical discharges to the right hemisphere and vertex of the nondominant side or bitemporally.	[20]
Magnetic Seizure Therapy (MST)	Like ECT, MST employs a powerful, frequently discharged magnet to induce localized synchronous activity in the cortical region of interest, which can spread to adjacent brain regions and produce a generalized seizure.	[21]
Theta burst stimulation (TBS)	TBS is a new type of rTMS that approximates the brain's endogenous theta frequency to better target and promote cortical plasticity.	[11]
Accelerated rTMS (arTMS)	arTMS is a form of treatment that utilizes many sessions per day, slightly lowering the overall amount of time spent undergoing stimulation over a couple of days.	[22]

We aim to explore the published literature regarding the drug and non‐drug treatment approaches for TRD. The intention is to compile and provide this material in a manner that would be useful to medical practitioners, psychiatrists, and researchers in the field.

## 2 Challenges of Identifying Treatment‐Resistant Depression

Most individuals diagnosed with MDD demonstrate a lack of response to the currently prescribed conventional therapies. In the realm of healthcare, the phenomenon of resistance evolution has been observed to occur in individuals who were once receptive to treatment. This process can manifest either as a gradual decline in health or a sudden shift towards a worsening illness trajectory [23]. According to numerous studies published in the literature, it has been found that the most prevalent definition for TRD in individuals with MDD is characterized by a minimum of two unsuccessful treatment attempts. Additionally, it is necessary to confirm that these previous treatments were administered at an appropriate dosage and for a sufficient duration [24].

The term pseudoresistance has been utilized to describe the lack of response to insufficient treatment due to inadequate duration or dosage of the prescribed antidepressant(s). It is essential to determine if any patient who exhibits an insufficient response to antidepressant treatment has received sufficient antidepressant treatment. Has the patient been administered antidepressant treatment for 8–12 weeks? Were the prescribed doses within the therapeutic range, as recommended by the healthcare professional? Has the patient adhered to the prescribed medication regimen? Have multiple doses been omitted? These inquiries must be addressed to properly assess the situation and eliminate the potential for pseudoresistance [25, 26].

Doctors must evaluate their patients' treatment adherence before concluding that their condition is resistant to therapy. Between 20% and 50% of people reportedly do not take their medications as prescribed. Cognitive impairments and feelings of worthlessness and hopelessness are strongly associated with this condition. Consistent cognitive behavior therapy (CBT) and regular doctor visits may help patients stick with their treatment plans. Adherent individuals, particularly those on tricyclic antidepressants, may benefit from measuring serum medication levels [27, 28, 29].

## 3 Recent Therapeutic Interventions

### 3.1 Augmentation

The findings presented in the existing randomized clinical trials (RCTs) examining the effects of antipsychotics as an augmentation therapy in TRD suggest the potential efficacy and advantages of utilizing second‐generation antipsychotics (SGAs), specifically aripiprazole and quetiapine, for TRD treatment. However, it is important to note that these results are still in the early stages and require further investigation. Hence, one may plausibly postulate that supplementing antidepressants with SGAs may be a robust approach to managing TRD [30].

In a clinical trial, the patient was administered a low dose of prazosin as an adjunctive therapy, which resulted in notable enhancements in symptoms related to post‐traumatic stress disorder (PTSD) and TRD. This case report highlights the rapid antidepressant effect of prazosin in a female adolescent patient with TRD who also suffers from PTSD. Prazosin has positively affected your well‐being, improving depression symptoms, sleep quality, suicidal ideation, and cognitive function. Prazosin was well tolerated by the patient, with no apparent adverse effects observed [31].

A study found that adding lithium to ketamine in animals that did not respond to antidepressants enhanced antidepressant‐like behavioral responses when subjected to stress. Additionally, this combination led to increased insulin release in the peripheral regions and specific insulin signaling in the prefrontal cortex [32]. Another study demonstrated that the addition of pramipexole to the therapy of individuals with TRD without psychotic symptoms, whether they have unipolar or bipolar disorder, is both beneficial and safe over a period of 24 weeks. The study found that patients who exhibited high levels of treatment adherence experienced more favorable results. Additionally, it was seen that those with more severe depression symptoms at the beginning of the study had lower rates of achieving remission [33].

Existing literature has provided evidence of a limited advantage associated with using folate supplementation for managing treatment‐resistant depression in adults. However, there is a scarcity of research investigating the efficacy of this intervention in the juvenile population. A case series examines a group of adolescents, with a mean age of 14.4 ± 2.8 years, who were diagnosed with treatment‐resistant depression and were provided supplementary l‐methylfolate (LM). Most individuals, specifically 80%, exhibited notable enhancements in symptoms related to depression, anxiety, and irritability. These findings indicate that using LM as a supplementary approach to antidepressant therapy could be a viable and successful for addressing treatment‐resistant depression in children [34].

A published article by Rittmannsberger details the addition of 50 mg amisulpride (AMS) to antidepressant therapy in seven individuals with depression at various levels of treatment resistance, one of whom had recurring short depression. Most patients' psychopathology significantly improved with AMS augmentation. Prolactin level increases and sporadic weight gain were the only negative effects. Most of the time, improvements happened quickly after just 1–2 weeks of treatment. A sudden and severe exacerbation of depression symptoms in some patients that resembled a withdrawal syndrome followed the reduction or stop of AMS [35].

### 3.2 Combination of Therapies for TRD

Transcranial magnetic stimulation (TMS) and ketamine infusion are two well‐known depression treatments. Best et al. investigated the clinical advantages of combining them for patients with treatment‐resistant depression. The findings have substantiated the sustained remission over an extended duration (specifically, 2 years postintervention) attained through combination TMS in conjunction with ketamine (CTK) for individuals afflicted with depressive syndromes. It is significant to acknowledge that CTK has demonstrated prolonged effectiveness in individuals with treatment‐resistant depression in cases where alternative interventions have proven unsuccessful. The enhanced patient adherence to the CTK protocol, resulting from the reduced number of treatment sessions, contributes to the successful management of treatment‐resistant depression and the achievement of sustained remission [36].

While both repetitive transcranial magnetic stimulation (rTMS) and intravenous ketamine have demonstrated efficacy in managing TRD, it is important to note that some patients may still exhibit resistance to these treatment options. In their research, Elkrief et al. present the case of a patient with bipolar TRD. The patient responded inadequately to initial and subsequent drug and psychological therapy. Following the limited response to both rTMS and ketamine as standalone treatments, a combination protocol involving rTMS and ketamine was implemented, resulting in comprehensive and enduring remission [37].

According to Liu et al. combining esketamine with an antidepressant demonstrates a clear therapeutic impact in managing TRD, leading to quick amelioration of depressive symptoms, enhanced quality of life, and increased patient satisfaction. However, it is worth noting that there may be minor adverse effects associated with this treatment approach [38].

### 3.3 Complementary and Alternative Medicines for TRD

Preliminary findings suggest a potential deficiency of EPA (ethyl‐eicosapentaenoate) in individuals experiencing depression, with indications that EPA treatment may be efficacious in alleviating depressive symptoms, particularly in individuals who are resistant to conventional treatments and have melancholy features. In addition, it has been observed that the EPA compound has the potential to augment the efficacy of antidepressant medications, particularly selective serotonin reuptake inhibitors (SSRIs), which are substrates of the p‐glycoprotein (p‐gp) transporter responsible for actively removing these drugs from the intracerebral space through the blood‐brain barrier [39]. Krawczyk and Rybakowski claimed that most individuals with treatment‐resistant depression saw significant symptom improvement after adding omega‐3 fatty acids to their regular antidepressant medication. When fatty acids were given, the clinical benefit was about the same as when lithium and lamotrigine were used to boost the effects of therapy. High amounts of fatty acids had no major negative effects [40].

### 3.4 Novel Therapeutics

A recent randomized, double‐blind clinical trial with 233 patients with TRD provided evidence for the therapeutic effectiveness of a single dose of psilocybin. Psilocybin significantly lowered depression scores in this phase 2 trial involving people with TRD [41]. A recent study has discovered that administering a single, open‐label dose of psilocybin at 25 mg has shown promising results for individuals with TRD. This study, conducted alongside selective serotonin reuptake inhibitors (SSRIs), revealed that participants reported a positive and tolerable experience. These findings provide valuable support for the continued exploration and development of psilocybin as a potential treatment option for individuals suffering from TRD when used with psychological support. This innovative design has the potential to significantly decrease the number of resources needed for administration while also offering a more convenient option for future research or therapy. Recent findings have indicated potential value in exploring the use of psilocybin as a supplement to antidepressant drugs [42]. Another study showed that psilocybin has a distinct pharmacological activity than standard medications for depression (i.e., 5‐HT2A receptor agonism), suggesting that it may be an effective supplement to conventional treatments for depression [43].

Ketamine, a noncompetitive antagonist of the N‐methyl‐d‐aspartate (NMDA) receptors, has shown efficacy in MDD and TRD [44], providing credibility to the idea that glutamate plays a significant role in mood modulation. According to previous research by Ahmed et al. administering several ketamine infusions can potentially mitigate suicidal ideation (SI) and symptoms of depression, irrespective of any accompanying mental or behavioral disorders [45]. In addition to the above research, Tham et al. suggested that repeated subcutaneous (SC) ketamine treatments may be a viable and advantageous option for individuals with TRD, offering a safe, feasible, and successful alternative to intravenous (IV) ketamine infusions [46].

The S‐enantiomer of ketamine, esketamine, has received plenty of interest in studying psychological diseases. Notably, it has recently received approval from the FDA in the United States for its potential in treating depression, which has proven resistant to multiple trials of antidepressant medications [47]. According to clinical trial findings, intranasal esketamine (IN esketamine) has notable efficacy in alleviating symptoms associated with TRD in the short term. Furthermore, the reported adverse effects are often temporary and of mild‐to‐moderate severity [48]. Daly et al. have provided evidence that administering esketamine with an antidepressant, following an initial 16‐week treatment period, yields notable and clinically significant advantages in relapse prevention for patients with TRD. Furthermore, this study offers additional safety data that supports a favorable balance between the benefits and risks associated with long‐term treatment [49].

The unique general anesthetic propofol interacts with N‐methyl‐d‐aspartate and gamma‐aminobutyric acid receptors, which Carhart‐Harris et al. suspected had antidepressant effects. A preliminary open‐label trial examined deep propofol anesthesia's feasibility, acceptability, and efficacy for treatment‐resistant depression. Ten healthy individuals aged 18–45 with moderate‐to‐severe medication‐resistant depression got 10 propofol infusions. Twenty electroconvulsive therapy patients were compared to self‐rated depression levels. All patients tolerated propofol. No major incidents happened. Self‐rated depression improved similarly with propofol and ECT. Comparing all metrics showed that high‐dose propofol treatment is possible and well tolerated by healthy people with treatment‐resistant depression. Propofol has quick, long‐lasting antidepressant effects like ECT but fewer side effects [50].

### 3.5 Deep Brain Stimulation for TRD

Using deep brain stimulation (DBS) on several components within the brain's reward system presents a potentially effective treatment approach for TRD patients. Recent studies have shown that DBS targeting the habenula (HB), a component of the brain's anti‐reward system, has potential to reduce depressive symptoms in individuals with TRD [51]. In a report, Kennedy et al. show how DBS to the subcallosal cingulate gyrus has helped 20 people with depression that has not responded to other treatments. After the first year of DBS treatment, patients returned once a year and then once more for an endpoint assessment of depression severity, functional outcomes, and side events. Until the most recent checkup, the patient's physical and social skills had steadily improved. According to the results of this study, DBS is still a viable option for treating depression that has not responded to other methods [52].

In a preliminary study with a few patients, DBS delivered to the nucleus accumbens showed antidepressant effects that persisted for up to 4 years. The observed effects at this specific stimulation site included anxiolytic properties and improved social functioning [53]. In a 6‐month double‐blind, sham‐controlled experiment, it was verified the safety and viability of subcallosal cingulate DBS as a treatment for TRD, although it did not demonstrate statistically significant antidepressant effects [54].

According to the preliminary findings of a study, there is a possibility that treatment‐resistant major depressive illness could benefit from bilateral stimulation of the superolateral branch of the medial forebrain bundle. A higher proportion of the population responded at lower stimulation intensities than in earlier trials, and the beginning of the antidepressant's efficacy was noticed within a short time [55]. According to Bergfeld et al. applying deep brain stimulation targeting the ventral anterior limb of the internal capsule (vALIC) yielded noteworthy reductions in depressive symptoms among 10 out of 25 patients, with favorable tolerability observed. The findings from the randomized crossover design support the notion that the ventral anterior limb of the internal capsule DBS (vALIC DBS) leads to decreased symptoms, as opposed to a sham intervention [56].

### 3.6 Vagus Nerve Stimulation for TRD

Stimulation of the vagus nerve, often known as VNS, has effectively managed TRD. VNS's neuroplastic effects on the hippocampus may explain its long‐term and permanent efficacy in the described case series of patients with severe multiepisode chronic depression. Findings indicated VNS as a feasible therapy option for TRD due to its general tolerability [57]. In a 5‐year study conducted in the TRD registry, Kumar et al. evaluated the durations of response obtained with adjunctive vagus nerve stimulation (VNS + TAU) versus therapy as usual (TAU) alone in patients with TRD. Data from 271 participants who received TAU and 328 who received VNS in addition to TAU were thoroughly studied. VNS therapy, when combined with TAU, has been observed to result in a swift onset and increased probability of response in cases of severe TRD. Additionally, this combined approach offers a greater sustainability of the response compared to TAU alone [58].

Additionally, a separate study has shown that adjunctive VNS has yielded more favorable results in terms of effectiveness and mortality when compared to treatment as usual alone. This study was conducted over a span of 5 years and focused on patients with chronic, severe treatment‐resistant depression. No evidence‐based therapy options were available for the patient population investigated in that experiment [59]. Kucia et al. used VNS therapy and stable medication for six patients with TRD. The trial was open‐label, uncontrolled, and retrospective. Only the VNS parameters were adjusted during the first 3 months, while the pharmaceutical treatment remained the same. However, during the subsequent 9 months, the drug and the VNS dosing parameters were adjusted based on the patient's clinical needs. After analyzing the results from the study, it was observed that patients with TRD benefited from VNS therapy, and its effectiveness improved over time [60].

### 3.7 Theta Burst Stimulation for TRD

One of the biological antidepressant treatments available is theta burst stimulation (TBS). The study conducted by Cheng et al. yielded results indicating that the application of left prefrontal intermittent theta burst stimulation (iTBS) was more effective in treating medication‐resistant depression compared to right prefrontal continuous theta burst stimulation (cTBS) or a combination of both techniques [61].

Using a comprehensive suicide risk assessment scale, Desmyter et al. wanted to determine whether or not accelerated iTBS increased the risk of suicide in a sample of treatment‐resistant unipolar depressive patients. Additionally, they wanted to determine whether or not iTBS was safe. A study comprising a cohort of 50 patients with therapy‐resistant depression who had not been treated with antidepressant medication administered a rigorous regimen of accelerated iTBS. The treatment was implemented using a randomized, sham‐controlled crossover design. Following the administration of 20 sessions of iTBS within a span of 4 consecutive days, it was observed that patients exhibited a favorable tolerance towards the iTBS protocol. A noteworthy reduction in the BSI (Beck Scale of Suicide Ideation) score was observed throughout the study, irrespective of the type of stimulation administered (active or sham) and independent of the response to depression treatment. Furthermore, no exacerbation of suicidal ideation was detected during the investigation [62].

According to the findings of Li et al. their randomized sham‐controlled study provides evidence supporting the claim that active theta‐burst stimulation is a form of repetitive transcranial magnetic stimulation (rTMS) that is well‐tolerated by individuals. Moreover, the study suggests that this particular form of stimulation exhibits favorable antidepressant efficacy, especially among participants who fall within a specific range of treatment resistance [63]. A pilot study by Fitzgerald et al. concluded that iTBS exhibits considerable potential as a TMS protocol for implementation within intensive therapeutic regimens. They also added that the administration of intensive TBS had demonstrated favorable tolerability and yielded clinical outcomes comparable to those observed with a conventional regimen of rTMS therapy [64].

Recent research has shown that accelerated iTBS (aiTBS) is safe and may be more effective than standard iTBS. Acute inpatient mental care settings may be able to accommodate aiTBS treatments due to their shorter length. The response and remission rates for TRD patients treated with aiTBS may be higher than those treated with the regular iTBS procedures approved by the FDA. Patients at risk for TRD now have more treatment options due to aiTBS's shorter time course, which makes the therapy more accessible in the inpatient setting [65].

### 3.8 Electroconvulsive Therapy for TRD

With a long history of success and modifications to treat refractory and severe depression with a high remission rate after numerous treatments, electroconvulsive therapy (ECT) is a significant but neglected treatment option [66]. In their study, Zhand et al. observed notable clinical improvement in most participants. Specifically, out of the 13 adolescents who underwent ECT for managing treatment‐resistant depression, 10 individuals (equivalent to 77% of the sample) experienced a clinically significant amelioration. This finding indicates that ECT holds promise as a viable therapeutic approach for refractory depression in a carefully chosen subset of adolescent patients [67].

In a large, countrywide study of ECT for unipolar, nonpsychotic depression, 65.9% of TRD patients and 75.9% of non‐TRD patients reported significant improvement [68]. Delmonte and colleagues have provided evidence highlighting the crucial significance of ECT in treating TRD. During their investigation, they took those patients who experienced persistent and intense episodes, which persisted for less than 1 year despite undergoing unsuccessful attempts at pharmacological intervention. ECT effectively facilitated rapid improvement in their clinical trajectory, devoid of any discernible untoward effects. The ECT demonstrated comparable relapse rates to conventional pharmacological treatment [69].

### 3.9 Magnetic Seizure Therapy for TRD

The early data for the antidepressant effects of magnetic seizure therapy, a novel convulsive therapy, is encouraging. Treatment‐resistant bipolar depression benefited greatly from magnetic seizure therapy, with no noticeable effect on cognitive function [70]. Preliminary data from the Kayser et al. trial showed that Magnetic Seizure Therapy (MST) has the same antidepressant effect as ECT. In both therapy groups, the antidepressant response was statistically significant and of comparable size without showing any cognitive side effects. The characteristics of seizures generated by MST and ECT were comparable, particularly in terms of ictal activity and postictal suppression. They concluded that if efficacy and safety are proven in bigger clinical trials, MST may be a viable alternative to ECT [71]. In their investigation of amnesia‐related side effects, Kayser et al. concluded that MST does not generate significant anterograde and retrograde amnesia [72].

The study conducted by Kayser et al. provided evidence of the great effectiveness of MST as an antidepressant and anti‐anxiety treatment. Furthermore, the study revealed that this efficacy is linked to specific metabolic alterations in brain regions that play a significant role in depression. Therefore, it may be concluded that MST offers a viable treatment alternative for those unresponsive to alternative therapeutic interventions for depression, demonstrating effectiveness, tolerability, and safety [73].

### 3.10 Psychotherapy for TRD

Exploring psychotherapeutic interventions for managing TRD is a nascent scientific research endeavor. The Cognitive Behavioral Analysis System of Psychotherapy (CBASP) is a unique therapeutic framework for treating chronic depression. The study conducted by Sayegh et al. involved the participation of all patients subjected to 12 group therapy sessions utilizing the CBASP. Before the group sessions, each patient also underwent two to four individual preparatory sessions. The study indicated that implementing CBASP group treatment yielded favorable patient outcomes. The findings of this study indicate that patients saw notable reductions in symptoms of depression and reliance on emotion‐oriented coping strategies. Additionally, they showed improvements in general social adjustment and interpersonal self‐efficacy compared to their initial levels before therapy. The findings from this pilot trial are promising and provide evidence for conducting additional research on the efficacy of CBASP group treatment with a control group [74].

Fonagy et al. conducted a randomized controlled trial to determine whether or not patients with long‐standing major depression who had failed at least two treatments and were considered to have treatment‐resistant depression would benefit from the addition of long‐term psychoanalytic psychotherapy (LTPP) to treatment‐as‐usual (TAU) according to UK national guidelines. The LTPP group exhibited significant gains in social adjustment and observer‐based and self‐reported depression. The aforementioned data indicate that Long‐term Psychopharmacotherapy may enhance the overall prognosis of individuals diagnosed with treatment‐resistant depression [75]. Guidelines for treating TRD should include an add‐on of psychotherapy to TAU in addition to pharmacological and neurostimulatory treatments, according to a meta‐analysis by van Bronswijk et al. [76].

## 4 Conclusion

The absence of consistency in the characterization of treatment‐resistant depression could potentially impact the management of patients. Various pharmacological, neuromodulation, psychotherapy, and novel therapeutic options are currently being explored as potential alternatives with promising outcomes. Augmentation or a combination of medications may be considered as viable options. Various forms of stimulation, including deep brain and theta burst stimulation and electroconvulsive therapy, have demonstrated promising results in research studies. Novel pharmacological agents, such as psilocybin and esketamine, hold great promise as potential alternatives for the future. Based on our assessment of the available evidence, various pharmacological and nonpharmacological treatment options have been formulated, as mentioned earlier. However, additional research is required to accurately diagnose, comprehend the underlying mechanisms, and effectively address treatment‐resistant depression to achieve long‐lasting improvement.

## Author Contributions

**Md. Rajdoula Rafe:** conceptualization, investigation, writing–original draft, methodology, writing–review and editing, formal analysis, supervision, data curation. **Abdul Waris:** writing–original draft, writing–review and editing, data curation, formal analysis. **Pranoy Saha:** writing–original draft, writing–review and editing, formal analysis, data curation.

## Conflicts of Interest

All authors have read and approved the final version of the manuscript. Md Rajdoula Rafe had full access to all of the data in this study and takes complete responsibility for the integrity of the data and the accuracy of the data analysis. The authors also declare no financial or nonfinancial conflict of interest.

## Transparency Statement

The lead author Md. Rajdoula Rafe affirms that this manuscript is an honest, accurate, and transparent account of the study being reported; that no important aspects of the study have been omitted; and that any discrepancies from the study as planned (and, if relevant, registered) have been explained.

## Data Availability

The authors have nothing to report.
